# Minimally invasive management of chronic venous insufficiency: A case report on combined radiofrequency ablation and sclerotherapy in an obese patient

**DOI:** 10.1016/j.ijscr.2025.111290

**Published:** 2025-04-12

**Authors:** M.A. La Marca, S. Bruno, E. Dinoto, R. Federico, F. Pecoraro, D. Mirabella

**Affiliations:** a Vascular Surgery Unit, AOUP Policlinico ‘P. Giaccone’, Palermo, Italy; b Department of Surgical, Oncological and Oral Sciences, University of Palermo, Italy

**Keywords:** Varicose veins, Hybrid treatments, Minivasive treatments, Obesity

## Abstract

**Introduction:**

Chronic venous insufficiency (CVI) affects a significant portion of the population, particularly impacting those with obesity. This condition leads to various symptoms, including leg discomfort and edema, contributing to work absenteeism. Traditional surgical procedures, like saphenous vein stripping and phebectomy, are increasingly supplanted by minimally invasive techniques, such as radiofrequency ablation (RFA) and sclerotherapy, which reduce invasiveness and associated complications, particularly beneficial for high-risk patients, including those with obesity.

**Case presentation:**

A 22-year-old male patient with a BMI of 41, suffering from severe varicose veins, hypertension, diabetes, and obstructive sleep apnea. The patient underwent simultaneous RFA and sclerotherapy after imaging confirmed significant venous incompetence but ruled out deep vein thrombosis. The procedure, performed under spinal anesthesia, resulted in successful obliteration of the great saphenous vein, with no postoperative complications.

**Clinical discussion:**

The literature highlights the advantages of endovascular techniques over traditional open surgery, particularly for patients with comorbidities that elevate surgical risks. Studies support the efficacy of combining RFA with sclerotherapy, showing improved outcomes and reduced recurrence rates. This combined approach minimizes complications and provides a quicker recovery, making it a suitable option for high-risk patients.

**Conclusion:**

Our experience and the findings of literature suggest that radiofrequency ablation paired with sclerotherapy is an effective treatment modality for managing CVI, particularly in patients with obesity.

## Highlights

• The treatment of varicose veins with not invasive techniques improves the outcome.
• The combination of radiofrequency and sclerotherapy is well-documented in literature.
• Obesity is a significant risk factor for recurrences of venous treatments.
• A combined approach improves the treatment outcomes for venous patients with obesity.
• The combined approach reduces operational risks for obese patients.

## 1 Introduction

Chronic venous insufficiency (CVI) affects approximately 10–60 % of population, with an incidence of about 2.6 % in women and 1.9 % in men. It is one of the primary causes of work absenteeism due to symptoms that commonly include leg discomfort, which manifesting as pain, heaviness, itching or burning sensations [1,2]. The main risk factors for CVI include age, gender, family history, and prolonged periods of sedentary or standing posture [3]. The issue of venous insufficiency is particularly significant in obese patients, who face unique challenges in both a surgical interventions and the management of pharmacological and elastic compression therapies [4]. According to the Clinical, Etiological, Anatomical, and Physiopathological (CEAP) classification, venous insufficiency can have primary, secondary, or congenital etiology (Table 1). The most common primary etiology is the loss of elasticity in the venous wall, which leads to the incompetence of unidirectional valves and subsequently causes venous hypertension. It is essential to consider post-thrombotic syndromes and congenital alterations, as superficial venous insufficiency may be an epiphenomenon that conceals more serious underlying conditions that should be addressed in therapy planning [5,6]. CVI is characterized by various stages, ranging from asymptomatic conditions, primarily aesthetic in nature, to severe, debilitating states with ulcerations that can affect large areas of skin tissue. [7] Traditional procedures such as saphenous vein stripping and phlebectomy are increasingly being supplanted by minimally invasive techniques, including Endovenous Laser Ablation (EVLA) and Radiofrequency Ablation (RFA), often in conjunction with sclerotherapy. This shift aims to minimize the invasiveness and complications associated with open surgery. Furthermore, these techniques can often be performed in a single session, making them suitable for patients who may not be candidates for conventional open surgery, such as those with obesity. In this report, we present a case of simultaneous treatment utilizing RFA and sclerotherapy in a patient with severe varicose veins and high-grade obesity (Body Mass Index >35). This work has been written in accordance with the SCARE criteria [8,9].

**Table 1:** CEAP classification.

Clinical	
C Class	Description
C0	No visible or palpable signs of venous disease
C1	Teleangectasieas or reticular veins
C2	Varicose Veins
C2r	Recurrent Varicose Veins
C3	Edema
C4	Changes in Skin and subcutaneous Tissue Secondary to Chronic Vein Disease
C4a	Pigmentation or Eczema
C4b	Lpodermatosclerosis or Atrophie Blanche
C4c	Corona Phebectatica
C5	Healed Venous Ulcer
C6	Active Venous Ulcer
C6r	Recurrent Active Venous Ulver


Etiological	
E Class	Description
Ep	Primary
Es	Secondary
Esi	Secondary - Intravenous
Ese	Secondary - Extravenous
Ec	Congenital
En	No Cause Identified


Anatomical			
A Class	Description		
As	Superficial		
	**Description**	**Old**	**New**
	Teleangectasiea	1	Tel
	Reticular Veins	1	Ret
	Great Saphenous Vein Above Knee	2	GSVa
	Great Saphenous Vein Belove Knee	3	GSVb
	Small Saphenous Vein	4	SSV
	Anterior Accessory Saphenous Vein	4	AASV
	Nonsaphenous Vein	5	NSV
Ad	Deep		
	Inferior Vena Cava	6	IVC
	Common Iliac Vein	7	CIV
	Internal ciac Vein	8	IIV
	External Iliac Vein	9	EIV
	Pelvic Veins	10	PELV
	Common Femoral Vein	11	CFV
	Deep Femoral Vein	12	DFV
	Femoral Vein	13	FV
	Popliteal Vein	14	POPV
	Crural (Tibial) Vein	15	TIBV
	Muscular Vein	16	MUSV
	Gastrocnemius Vein	16	GAV
	Soleal Vein	16	SOV
	Perforator		
	Thigh Perforator Vein	17	TPV
	Calf Perforator Vein	18	CPV
An	No Venous Anatomic Location Identified		


Pathophysiological	
P Class	Description
Pr	Reflux
Po	Obstruction
Pr,o	Reflux and Obstruction
Pn	No pathophysiology Identified

## 2 Case report

**Fig. 1:**
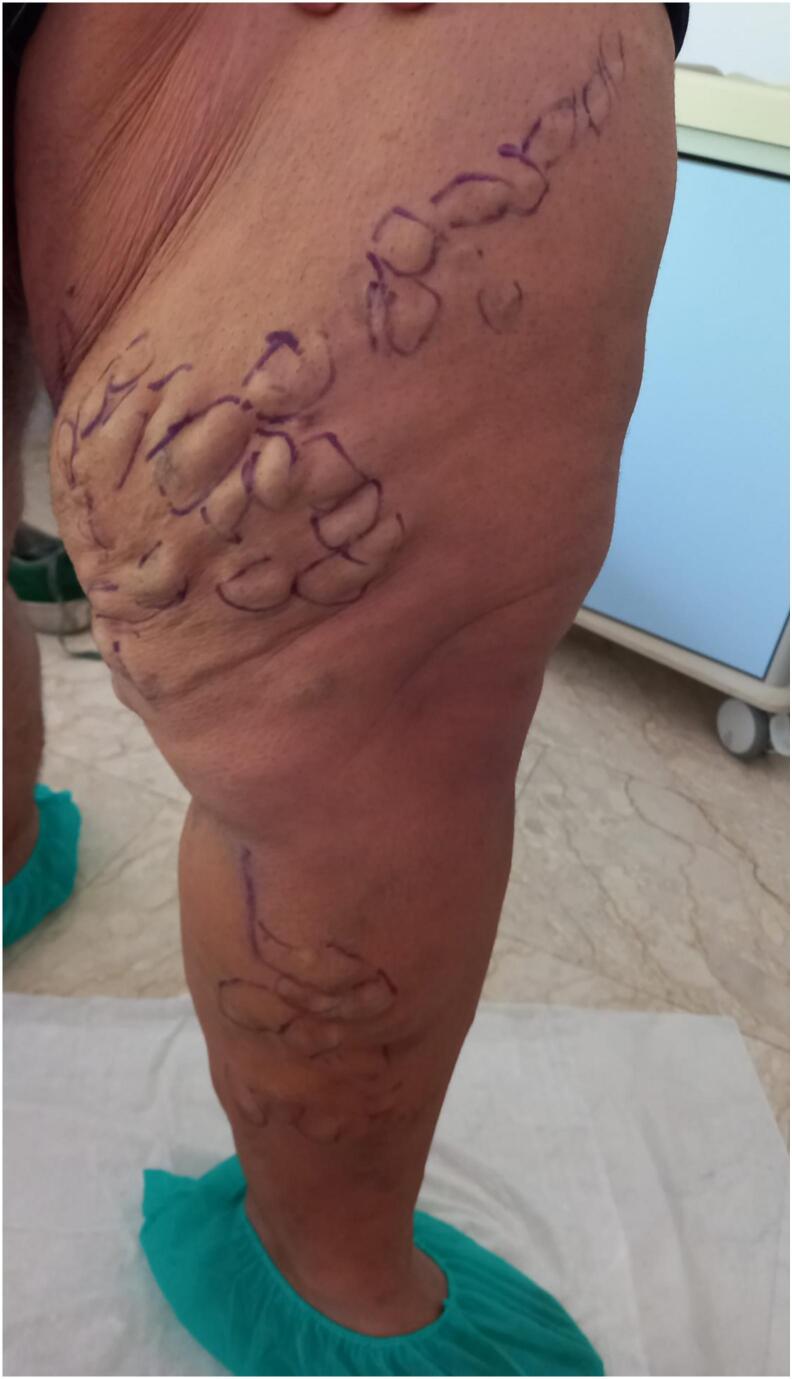
Varicose veins in obesity patient before procedure.

**Fig. 2:**
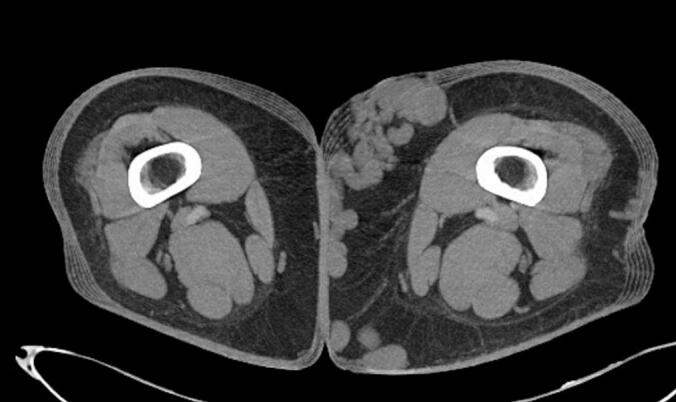
CT scan with widespread and significant varicose veins.

**Fig. 3:**
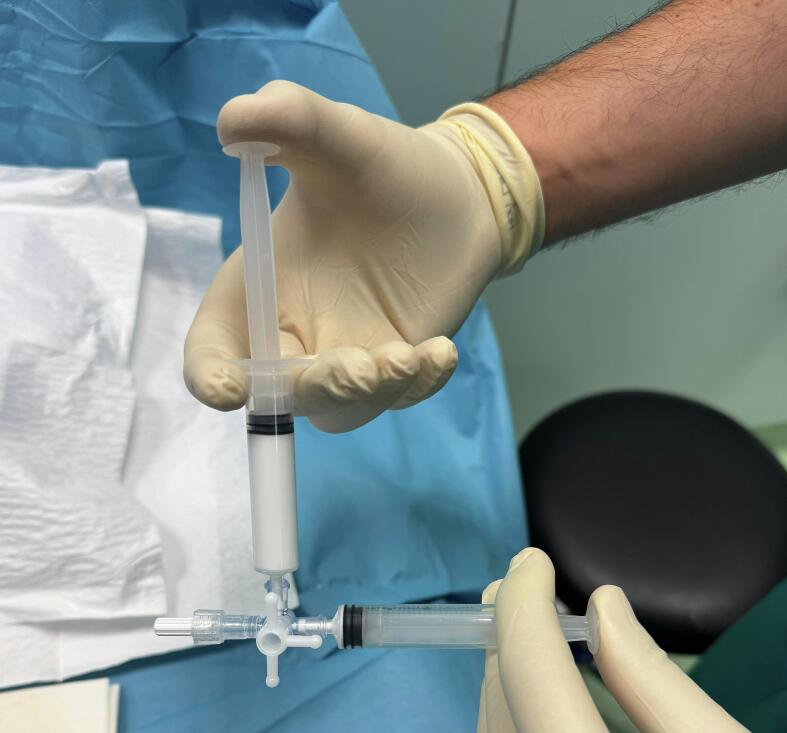
Preparation of foam 1 % Lauromacrogol.

**Fig. 4:**
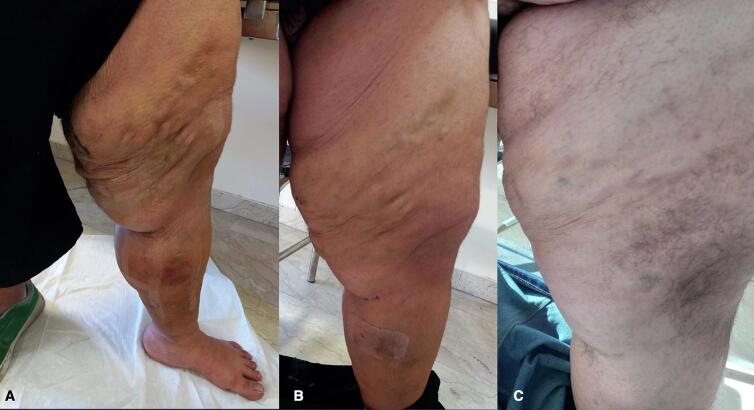
Outcome at 15 days (A), one month (B), 6 months (C).

A 22-year-old male patient with obesity, characterized by a Body Mass Index (BMI) of 41, presents with a variety of comorbid conditions, including hypertension, diabetes, obstructive sleep apnoea syndrome (OSAS), and anxiety-depressive disorder. He reports pain and a sensation of heaviness in his left lower limb, accompanied by significant edema (CEAP C4a) (Fig. 1). The patient denies any history of prior surgeries. Due to the patient's severe obesity, the results of the ultrasonography were inconclusive regarding the assessment of the deep venous system; however, they did indicate significant valvular incompetence at the saphenofemoral junction, extending to the distal third of the thigh along the great saphenous vein (GSV). To exclude the possibility of deep vein thrombosis (DVT) or other abnormalities that might contribute to venous hypertension, a computed tomography angiography (CTA) of the abdomen and lower limbs was performed. This imaging ruled out deep veins involvement and confirmed the presence of multiple venous ectasias affecting the superficial venous system (Fig. 2). The ectasias were most prominent in the soft tissues beginning at the anterolateral root of the left thigh and extending along the medial and posterior aspects of the middle third of the thigh, as well as the anteromedial side of the middle third of the leg. The extensive network of venous branches posed significant challenges for the treatment of varicose veins; any phlebectomies would require multiple incisions, therapy increasing the risk of infection. Given the diameters of the saphenous vein (maximum diameter of 8.9 mm in the distal third of the thigh and 15.7 mm at the saphenofemoral junction) and its relatively straight course, the patient was identified as a suitable candidate for saphenectomy, combined with RFA and sclerotherapy of the collateral veins. Taking into account the patient's size, limited cooperation due to anxiety-depressive syndrome, and the OSAS which could heighten the risk associated with increased intraoperative sedation, it was determined that performing the procedure under spinal anesthesia would be preferable. A 7 French introducer was inserted at the proximal third of the left leg. A radiofrequency device (vienCLEAR - RF Medical, Seoul, Korea) was introduced and positioned 2.5 cm from the saphenofemoral junction. To facilitate the adherence of the probe to the walls of the GSV, a tumescent solution totaling 250 ml was infused along the entire length of the vein. After ultrasound-guided confirmation of the correct placement of the guide tip, the GSV was ablated. The catheter was then connected to a generator that delivers radiofrequency energy upon activation. Each 7-cm segment of the vein was heated for 20 s before the energy supply automatically shut off. The catheter was then manually advanced to the next marker, and the heating process was repeated for subsequent segments. The proximal 7-cm segment of the GSV underwent two 20-s energy cycles, while the other segments were treated with either one or two cycles. For each 20-s cycle, the temperature required to reach 120 °C within 5 s of activation. If this temperature was not achieved within that timeframe, the segment underwent an additional 20-s treatment cycle. Post-ablation ultrasound check confirmed the complete obliteration of the GSV while ensuring the patency of some collateral veins responsible for extra-saphenous varicosities. The introducer was subsequently removed. To prevent any potential early recanalizations of the GSV due to the permeability of the saphenous collaterals, treatment continued with the Ultrasound-guided injection of 3 ml of 1 % Lauromacrogol foam into the venous collaterals of the proximal third of the leg (1 ml) and the middle third of the thigh (2 ml), followed by a dressing and elastic compression bandage (Fig. 3). The postoperative course was smooth, with the patient reporting no significant pain or swelling and 1 g paracetamol tablets were administered as needed. The patient was discharged home the following day. Follow-up appointments were scheduled at 15 days, 1 month and 6 months post-procedure, and ultrasound assessments indicated no complications or recurrences, allowing the patient to return to work without symptoms (Fig. 4).

## 3 Discussion

The prevalence of lower limb venous diseases and obesity is increasing. Numerous studies have shown that being overweight is a contributing factor to CVI, which can lead to the early onset of ulcerations. [[10], [11], [12]]. Surgical interventions on the superficial venous system typically carry a low operative risk; however, a patient's comorbidities can elevate the risks associated with these procedures. High ligation and stripping of GSV are usually conducted in an operating room under spinal or general anesthesia. Whenever possible, major forms of anesthesia should be avoided to reduce complications related to anesthetics, such as allergic reactions, damage to teeth during intubation, and post-operative nausea and vomiting [13]. Currently, treatment options for CVI include endovascular techniques such as RFA and EVLA, sclerotherapy and surgical therapies. Studies have demonstrated the effectiveness and safety of endovascular procedure compared with traditional open treatment [14]. The Society for Vascular Surgery and the American Venous Forum recommend endovascular techniques as the primary choice for treating superficial venous disease due to their reduced pain, lower morbidity, shorter hospital stays and quicker return to work [15]. The literature indicates that obesity is a significant risk factor for complications associated with any type of surgical intervention due to increased risks of cardiovascular, respiratory, and site infections [16,17]. Obese individuals are 47 % more likely to experience CVI compared to those with a normal body mass index. Previous research has indicated that changes in venous biomechanics, resulting from venous return obstruction linked to increased abdominal adipose tissue, contribute to a higher risk of cardiovascular disease in obese patients. Additionally, individuals with obesity often struggle with adhering to compression therapy due to difficulties in donning or wearing compression stockings, which may be influenced their body habitus. This can lead to poor compliance with compression therapy and diminish the clinical response following venous treatments. Moreover, it has been shown that BMI adversely affects the CEAP clinical classification, pain levels, and quality of life, independently of venous reflux. BMI has also been identified as a significant factor associated with less improvement in the revised Venous Clinical Severity Score after venous treatment [18]. Rodriguez-Avecedo et al. identified a BMI >30 as a potential criterion for distinguishing between patients at increased risk of recanalization and those who are not [19]. Other causes of early recanalization, as reported by Bissaco et al., may be attributed to specific sources of reflux, such as anterior or posterior accessory saphenous veins and perforating veins, irrespective of the primary ablation treatment [20]. Consequently, opting for a less invasive technique seems a logical choice, especially in overcoming the potential challenges posed by open techniques. The combination of multiple minimally invasive techniques may enhance the success rate and sustainability of treatment outcomes [21]. Ultrasound-guided foam sclerotherapy, combined with RFA closure, represents a novel approach to treating CVI [22]. This technique generates thermal energy via a radiofrequency catheter, targeting the intima and collagen fibers of affected venous vessels. The resulting thermal coagulation effect damages endothelial cells, leading to degeneration, thickening, organization, contraction, and shedding [23]. This process helps narrow the venous lumen and thicken the venous wall, ultimately closing varicose veins through the formation of fibrotic cords and achieving the desired therapeutic outcome [24]. The ultrasound-guided sclerotherapy, performed during the thermal ablation procedure, effectively eliminates the presence of feeding collateral veins that could supply the saphenous vein, one of the risk factors for recanalization. This minimally invasive procedure does not require stripping to eliminate the diseased venous vessels; instead, it occludes venous blood flow under local anesthesia to produce the therapeutic effect. This approach alleviates pain and minimizes associated complications, making it more acceptable to patients [25]. Numerous positive experiences have emerged from the combination of these two techniques. Zhao and Hongtao, in separate studies, demonstrated that the quality of life for patients treated with a combination technique was significantly higher than that of the control group six months after the operation (*P* < .05). This suggests that ultrasound-guided foam sclerotherapy combined with intracavity radiofrequency closure can effectively enhance the quality of life for patients with CVI by reducing the procedure length, postoperative bed rest time, hospital stays, and intraoperative blood loss when compared to traditional open treatment methods [21,26]. Memon et al. treated 102 cases with radiofrequency, of which 79 % underwent concurrent sclerotherapy. During the follow-up, 99 % of the treated patients experienced a permanent vessel occlusion [27]. Carrol et al. highlight in a literature review that out of 1453 unique citations, including 34 randomized clinical trials (54 articles), minimally invasive techniques yielded clinical results comparable to traditional surgery. Recurrence rates were slightly lower for EVLA, RFA, and Foam Sclerotherapy, especially during longer follow-up periods. Short-term pain was lower for Foam Sclerotherapy and RFA but higher for EVLA. All evaluated techniques reported better quality of life scores compared to stripping. Therefore, the differences between treatments were found to be negligible in terms of clinical outcomes, suggesting that the most cost -effective treatment is the one with the lowest cost. The total costs of minimally invasive treatments were lower and marginally more effective compared to stripping [28]. Among the limited studies focused on obese patients, Zottola et al. reported that those in higher obesity classes experience greater reductions in perceived symptoms during early follow-up after minimally invasive interventions. While obesity has been associated with increased severity of venous disease symptoms, obese patients can experience significant short-term relief following treatment, often achieving greater symptom improvement compared to non-obese patients, particularly when treated for milder disease presentations [12]. The methodological limitations are evident in the contraindications associated with these procedures. For instance, excessive tortuosity of the GSV can complicate RFA, while allergies to sclerosing agent can hinder sclerotherapy. Unfortunately, many pivotal studies have specifically excluded obese individuals [29]. Nevertheless, the substantial presence of obese patients suffering from venous insufficiency highlights the need for effective and potentially repeatable treatments. In our experience, the application of a combined technique in high-risk patient for complications and recurrence has allowed us to achieve the desired outcomes while minimizing risks.

## 4 Conclusions

Obesity is identified as a considerable risk factor for complications related to surgical interventions for CVI. The combination of RFA and simultaneous sclerotherapy is a technique that has shown a high rate of technical success and an improvement in postoperative quality of life. These findings emphasize the importance of customizing treatment strategies to align with individual patient profiles, especially for those who are obese or have other comorbidities. This customization is essential to ensure effective management of CVI while minimizing the risk of complications. Further research is needed to explore the long-term outcomes of combined minimally invasive techniques for CVI in obese patients, as existing literature frequently overlooks this demographic. The demand for accessible and effective treatment options for this population remains a critical issue in managing CVI.

## Credit authorship contribution statement

Conceptualization, MA.L., E.D. and D.M.; methodology, E.D., R.F.; software, M.A.L; validation, D.M., E.D. and F.P.; formal analysis, S.B. and E.D.; investigation, M.A.L.; resources, M.A.L., R.F.; data curation, E.D., D.M.; writing—original draft preparation, M.A.L.; writing—review and editing, E.D., D.M.; visualization, D.M., F.P.; supervision, F.P.; project administration, F.P. All authors have read and agreed to the published version of the manuscript.

## Informed consent statement

Written informed consent was obtained from the patient for publication of this case report and accompanying images. A copy of the written consent is available for review by the Editor-in-Chief of this journal on request.

## Ethical approval

All procedures followed were in accordance with the ethical standards of the Institutional Committee on Human Experimentation and with the Helsinki Declaration. According to the internal review board, the retrospective and anonymized nature of the study did not require medical ethical committee approval. This article does not require ethics committee approval due to the retrospective nature of the paper and the type of technique reported.

## Institutional review board statement

All procedures followed were in accordance with the ethical standards of the Institutional Committee on Human Experimentation and with the Helsinki Declaration. According to the internal review board, the retrospective and anonymized nature of the study did not require medical ethical committee approval.

## Guarantor

ETTORE DINOTO.

## Funding

This research received no external funding.

## Declaration of competing interest

The authors declare no conflict of interest.

## Data Availability

The data presented in this study are available on request from the corresponding author.
